# A clinical study on the efficacy of Yiqi Huayu Jiedu decoction for reducing the risk of postoperative recurrence and metastasis of gastric cancer: Protocol for a multicenter, randomized, double-blind, placebo-controlled trial

**DOI:** 10.1097/MD.0000000000023417

**Published:** 2020-11-25

**Authors:** Cun-En Wu, Wei-Wei Xue, Yu-Wen Zhuang, Da-Wei Ding, Jin-Yong Zhou, Shen-Lin Liu, Rui-Ping Wang, Peng Shu

**Affiliations:** a Department of Oncology, Affiliated Hospital of Nanjing University of Chinese Medicine, Jiangsu Province Hospital of Chinese Medicine; b Traditional Chinese Medicine Department, Jinling Hospital, School of Medicine, Nanjing University; c Central Laboratory, Affiliated Hospital of Nanjing University of Chinese Medicine, Jiangsu Province Hospital of Chinese Medicine, China.

**Keywords:** gastric cancer, multicenter randomized double-blind placebo-controlled trial, recurrence and metastasis, traditional Chinese medicine, Yiqi Huayu Jiedu decoction

## Abstract

**Background:**

Gastric cancer (GC) is one of the top 10 malignant tumors worldwide and poses a great threat to human life and health, the prevention and treatment of which has become the focus and difficulty of medical research. With its unique advantages, traditional Chinese medicine (TCM) is widely used in the prevention and treatment of postoperative recurrence and metastasis of GC as well as the improvement of patients’ quality of life. The aim of this study is to elucidate the curative effect and the underlying mechanism of Yiqi Huayu Jiedu (YQHYJD) decoction.

**Methods/design:**

This is a prospective, multicenter, randomized controlled trial continuing 3 years. Two hundred ninety-eight eligible patients will be randomly divided into 2 groups, the chemotherapy combined with placebo and the chemotherapy combined with YQHYJD group at a ratio of 1:1. All patients will receive the treatment for 6 months and follow up for 3 years. The primary outcomes are disease-free survival, and 1-year, 2-year, 3-year progression-free survival rate, while the secondary outcomes are tumor makers, TCM syndrome score, quality of life score, overall chemotherapy completion rate, intestinal flora diversity test, immune function (T, B lymphocyte subsets and NK cells) test. The Security index includes blood, urine and stool routine, electrocardiogram, liver function (ALT), and renal function (BUN, Scr). All of these outcomes will be analyzed at the end of the trial.

**Discussion:**

This research will provide the valuable evidence for the efficacy and safety of Yiqi Huayu Jiedu decoction in postoperative GC. Furthermore, it will be helpful to form a higher level of evidence-based medical basis for TCM in the treatment of GC recurrence and metastasis.

**Trial registration:**

ChiCTR2000039038

## 1 Introduction

Gastric cancer (GC) is one of the top 10 malignant tumors in the world, ranking 6th in incidence rate and 2nd in mortality rate.^[1]^ China has a high incidence of GC, accounting for more than 40% of the world's confirmed cases. About 670,000 patients of GC are diagnosed, resulting in nearly half a million deaths each year.^[2]^ The 5-year survival rate of early GC is nearly 95%, while the 1-year recurrence and metastasis rate after surgery is about 50%, and the rate of 2-year is as high as 70%.^[3]^ Worsely, due to the atypical symptoms and low diagnostic rate of early GC, more than 90% of patients were diagnosed with locally advanced or metastatic GC at the time of initial diagnosis.^[4]^

Although intravenous chemotherapy, targeted therapy, and immunotherapy can control the lesions to a certain extent, they are all inevitable to the appearance of drug resistance, toxic and side effects, which seriously affect the prognosis and quality of life. Therefore, reducing the risk of recurrence and metastasis is one of the effective strategies to improve the comprehensive efficacy and prognosis of patients with GC. traditional Chinese medicine (TCM) plays a unique role in enhancement of immune function, detoxification, and synergism of chemoradiotherapy.^[5]^ In addition, it is reported to be benefit to resistance to recurrence and metastasis, prolongation of patients’ survival, and improvement of quality of life.^[6]^

The Yiqi Huayu Jiedu decoction is a traditional Chinese herbal formulation based on years of clinical experience established by Professor Shen-lin Liu, the national famous Chinese Physician in Affiliated Hospital of Nanjing University of Chinese Medicine. YQHYJD decoction consists of 10 kinds of Chinese herbal medicine shown in Table 1. Our previous research has revealed that YQHYJD decoction can suppress the invasion and metastasis of GC cells, the mechanism of which may be related to TGF-β/Smad pathway.^[7]^ The present project takes YQHYJD decoction or placebo combined with chemotherapy, to evaluate the efficacy of this prescription in the treatment of GC metastasis and recurrence, so as to form a treatment scheme suitable for clinical promotion and provide a higher level of evidence-based medical basis for TCM in the prevention and treatment of postoperative recurrence and metastasis in GC.

**Table 1:** Yiqi Huayu Jiedu decoction (YQHYJD) composition.

Chinese name	Latin name	Doses (g)
Sheng Huang Qi	Astragalus Root	45
Dang Shen	Codonopsis Pilosula	15
Chao Bai Zhu	Rhizoma Atractylodis Macrocephalae	10
Chao Bai Shao	Radix Paeoniae Alba	10
Dang Gui	Angelica Sinensis	10
Chen Pi	Pericarpium Citri Reticulatae	10
San Leng	Rhizoma Sparganii	20
E Zhu	Curcuma Zedoary	20
Shi Jian Chuan	Salvia Chinensis	30
Bai Hua She She Cao	Oldenlandia Diffusa	30

## 2 Methods/design

### 2.1 Design

**Figure 1:**
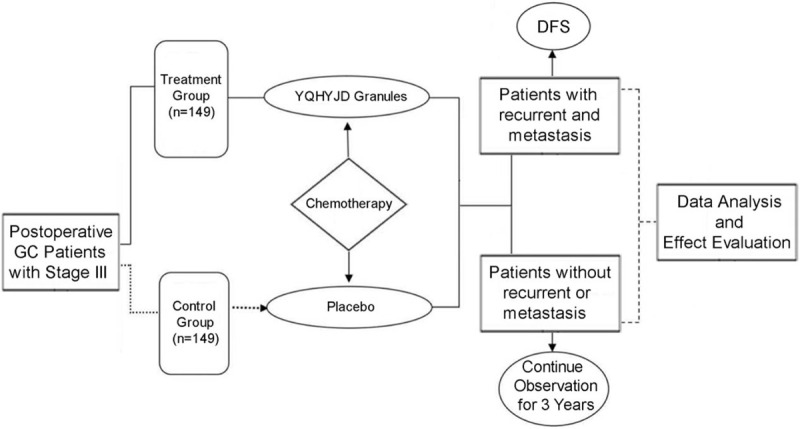
The research flowchart. The flowchart of enrollment, interventions, follow-up visits, and analysis.

This research is designed as a prospective, multicenter, randomized, double-blind, placebo-controlled trial lasting up to 3 years in 11 hospitals, including Affiliated Hospital of Nanjing University of Chinese Medicine, Guang’anmen Hospital, China Academy of Chinese Medical Sciences, Hubei Provincial Hospital of TCM, Guangdong Provincial Hospital of Chinese Medicine, Yueyang Hospital of Integrated Traditional Chinese and Western Medicine, Shanghai University of Traditional Chinese Medicine, First Teaching Hospital of Tianjin University of Traditional Chinese Medicine, Nanjing Drum Tower Hospital, Jiangsu Cancer Hospital, The First People's Hospital of Changzhou, Changzhou No. 2 People's Hospital, Zhangjiagang First People's Hospital. A total of 298 postoperative GC patients with stage III will be selected and randomly divided into 2 groups in a 1:1 ratio, with 149 in treatment group (YQHYJD granules + chemotherapy) and control group (placebo + chemotherapy), respectively. The protocol of our research has been approved by the Ethic Committee of Affiliated Hospital of Nanjing University of Chinese Medicine (Number 2020NL-121–02). The research flow chart is as shown in Figure 1.

### 2.2 Randomization and hiding

The professional statisticians generated random sequences by SAS (Proc Plan) statistical software. The 298 subjects were divided into the treatment and the control group in a 1:1 ratio (149 patients in each group). The random seeds and random sequences are kept as confidential data in an opaque envelope, and the emergency envelope is kept in a third party for safekeeping to ensure that patients, researchers, and outcome evaluators are not aware of the patient group before the blindness is revealed.

### 2.3 Blinding

In this study, double blind and single simulation will be performed. The treatment group is combined with YQHYJD granules on the basis of chemotherapy, while the control group is combined with YQHYJD simulants. The simulated agent particles are prepared with 2% original Chinese medicine and 2% bitter gourd extract, whose appearance, dosage form, weight, color, and odor are consistent with the experimental drugs. Both experimental drugs and the placebo are put into the same packaging according to the requirement of the double-blind trial.

### 2.4 Sample size

According to the the primary endpoints of previous clinical research,^[8]^ the disease-free survival (DFS) rate of postoperative GC patients is 50.42% in control group (chemotherapy alone) and 64.14% in treatment group (chemotherapy + Chinese medicine). The sample size is calculated via the following formula:

$$n=\frac{[u_{1-\alpha }\sqrt{p_{0}(1-p_{0})}+u_{1-\beta }{\sqrt{p_{T}(1-p_{T})}}^{2}}{{\left(p_{T}-p_{0}\right)}^{2}}$$

Considering 80% in the degree of assurance, and 2.5% in the class I error probability, the sample size calculation method of difference test of 2 independent sample rates by SPSS software is applied and 135 patients in each group are calculated. As a result, a total of 298 cases (149 for each group) were needed for 2 groups based on a shedding rate of estimated 10%.

### 2.5 Recruitment and consent

This study will be introduced to the interested parties through the release of official accounts, recruitment advertisements, etc, and the qualified subjects ought to be selected to sign the informed consent forms and enter the study. The recruitment notice and research brief shall be submitted to the ethics committee for review.

### 2.6 Inclusion criteria and exclusion criteria

#### 2.6.1 Inclusion criteria

(1) Pathological diagnosis meets the diagnostic criteria for GC, and TCM syndrome differentiation is weakness of spleen and stomach;
(2) Pathologically confirmed stage III and has implemented radical surgery;
(3) ECOG score ≤2, aged between 18 and 75;
(4) Estimated survival time is more than 6 months;
(5) Patients are volunteers and have signed informed consent. The process of obtaining informed consent complies with the GCP requirements.

#### 2.6.2 Exclusion criteria

(1) Patients with metastatic GC;
(2) Local recurrence or distant metastasis has been confirmed by histopathological or imaging examination;
(3) Women during pregnancy and lactation;
(4) Patients with severe primary cardiovascular, liver, renal, or hematological disease;
(5) Patients with serious primary heart, liver, lung, kidney, blood, or other severe diseases that affect their survival;
(6) Disabled patients (blind, deaf, dumb, mentally, mentally, or physically disabled) as prescribed by law;
(7) Suspected or existing history of alcohol or drug abuse;
(8) Other lesions that may reduce the possibility of enrollment or complicate the enrollment according to the judgment of the researcher, such as frequent changes of the work environment, that may easily lead to loss of follow-up;
(9) Allergic constitution, such as 2 or more drugs or food allergies; Or known to be allergic to the ingredients of YQHYJD decoction;
(10) Patients who are participating in clinical studies of other drugs.

### 2.7 Interventions

#### 2.7.1 The control group

One hundred forty-nine patients in the control group will be treated with chemotherapy combined with placebo. XELOX scheme, SOX scheme, or DS-1 scheme are selected according to the 2020 NCCN guidelines.

XELOX: Oxaliplatin 130 mg/m^2^ iv, on day 1; Capecitabine 1000 mg/m^2^ po, on days 1 to 14, twice daily, every 3 weeks

SOX: Oxaliplatin 130 mg/m^2^ iv, on day 1; S-1 40 mg/m^2^ po, on days 1 to 14, twice daily, every 3 weeks

DS-1: Docetaxel 40 to 50 mg/m^2^ iv, on day 1, day15; S-1 40 to 60 mg/m^2^ po, on days1–14, twice daily, every 4 weeks

#### 2.7.2 The treatment group

One hundred forty-nine patients in the treatment group will be treated with chemotherapy combined with YQHYJD granules. The chemotherapy regimens is designed to be the same as the control group. YQHYJD granules will be taken 1 bag twice daily, half an hour to 1 hour before breakfast and dinner with warm water. The drug was administered continuously for 6 months from the first day of the first cycle of chemotherapy.

### 2.8 Outcome measures

The time-points of assessment are listed in Table 2.

**Table 2:** Research schedule.

Time pointRecord contents		Cycle 1			Cycle 2			Cycle 3			Cycle 4			Cycle 5			Cycle 6			Months after chemotherapy							
	B/L	Week			Week			Week			Week			Week			Week			6	9	12	15	18	24	30	36
		1	2	3	1	2	3	1	2	3	1	2	3	1	2	3	1	2	3								
Informed Consent	×																										
Basic information	×																										
Gastroscope^∗^	×																			×				×			
CT/B-mode ultrasonography)^∗^	×												×							×	×	×	×	×	×	×	×
ECT^∗^	×																					×			×		×
Tumor markers	×			×			×			×			×			×			×	×	×	×	×	×	×	×	×
Quality of life score	×			×			×			×			×			×			×	×	×	×	×	×	×	×	×
TCM syndrome core	×			×			×			×			×			×			×	×	×	×	×	×	×	×	×
Overall chemotherapy completion rate	×			×			×			×			×			×			×								
Detection of intestinal flora diversity	×						×						×						×								
Detection of immune function	×						×						×						×								×
Blood routine	×			×			×			×			×			×			×	×	×	×	×	×	×	×	×
Hepatorenal function	×			×			×			×			×			×			×	×	×	×	×	×	×	×	×
ECG	×			×			×			×			×			×			×	×	×	×	×	×	×	×	×
Drug distribution	×	×	×	×	×	×	×	×	×	×	×	×	×	×	×	×	×	×	×	×							

#### 2.8.1 Primary outcomes

The primary outcomes include DFS and progression-free survival (PFS) rate at 1, 2, and 3 years. In detail, DFS means the time from randomization to disease recurrence or death due to disease progression. PFS means the time from randomization to disease progression or death due to any reason. DFS and PFS rates will be calculated at the end of the trial.

#### 2.8.2 Secondary outcomes

(1) Tumor markers: CEA, CA125, AFP, CA199, and CA153 will be examined at the baseline period, the third week of every cycle of chemotherapy, every 3 months from 6 months after chemotherapy, and every 6 months from 18 months after chemotherapy during the follow-up.
(2) TCM syndrome score: TCM syndrome containing emesis, numb, diarrhea, constipation, intolerance of cold, enuresis nocturna, amnesia, spontaneous perspiration, night sweat, thirst, and dental ulcer will be assessed at the baseline period, the third week of every cycle of chemotherapy, every 3 months from 6 months after chemotherapy, and every 6 months from 18 months after chemotherapy by questionnaires. The scores of each item ranged from 0 (asymptomatic) to 10 (worst severity imaginable).
(3) Quality of life score: physiological status, social/family status, emotional status, and functional status will be evaluated with FACT-G scale. The frequency will be the baseline period, the third week of every cycle of chemotherapy, every 3 months from 6 months after chemotherapy, and every 6 months from 18 months after chemotherapy.
(4) Overall chemotherapy completion rate: from the beginning to the end of the sixth cycle of chemotherapy, at the third week of every cycle.
(5) Detection of intestinal flora diversity: at the baseline period, the third week of the second, fourth, and sixth cycles of chemotherapy, respectively.
(6) Detection of immune function (T, B lymphocyte subsets, NK cells): at the baseline period, the third week of the second, fourth, and sixth cycles of chemotherapy, and the last follow-up period.

#### 2.8.3 Safety evaluation

The indicators of blood routine, urine routine, stool routine, electrocardiogram, liver function (ALT), renal function (BUN, Scr) will be detected periodically in order for the assessment of the safety of YQHYJD decoction.

### 2.9 Adverse reactions

(1) Researchers should explain to the subjects and ask them to truthfully reflect the changes in their condition after the medication.
(2) Researchers ought to pay close attention to adverse events or unexpected side effects (including symptoms, signs, and laboratory tests), analyze the causes, make judgments, and track the observation and records.
(3) For the adverse events that occurred during the study period, the symptoms, degree, time of occurrence, duration, treatment measures, and procedures should be recorded in the case report forms, and the correlation between the adverse events and the drugs should be evaluated.
(4) When adverse events are found, researchers can decide whether to suspend the observation according to the condition. Follow-up investigation should be carried out on the case of drug withdrawal due to adverse reaction, and the treatment process and results should be recorded in detail.
(5) When serious adverse events occur, researchers must take immediate measures to protect the safety of the subjects and report to the Ethic Committee of Affiliated Hospital of Nanjing University of Chinese Medicine.

### 2.10 Data analysis

For quantitative data, descriptive statistical analysis will be conducted with case number (N), Mean (Mean), standard deviation (Std) or median (Med), upper quartile (Q1), and lower quartile (Q3). For qualitative data, descriptive statistical analysis should be done by frequency table, percentage, or composition ratio. Comparison before and after treatment will be carried out through paired *t*-test or paired rank-sum test according to the data distribution type. The differences between groups were compared using *t*-test or rank-sum test for quantitative data and chi-square or rank-sum test for qualitative data. All statistical calculations were performed by SPSS statistical analysis software. Data should be analyzed with 2-sided test and *P* < .05 is considered as statistically significant.

## 3 Discussion

The incidence of gastrointestinal neoplasms in China is increasing year by year, among which GC ranks the first. The prognosis of GC is poor, whose five-year survival rate is only 20% to 30%. Nearly half of the patient are diagnosed with advanced stage and lost opportunity for operation. Even worse, patients are suffering from high risk of postoperative recurrence and metastasis, while the survival time of late gastric carcinoma is about to 10 months.^[9–10]^ During the last 3 decades, despite of the great progress in the mechanism, surgical technology as well as the research of anti-tumor drugs, the survival rate and quality of life of postoperative GC patients are not significantly improved.^[11–13]^

TCM is a treasure of ancient Chinese science. It may be one of the effective strategies to improve the efficacy of GC by seeking more suitable drugs for effective prevention and treatment of recurrence and metastasis. Increasing research has verified that TCM has an important potential value for bringing about better prognosis and reduced side effects.^[14–15]^ In addidtion, TCM is also able to improve clinical symptoms as well as living quality of patients with GC.^[16]^

In recent years, according to the disease characteristics and preliminary clinical analysis, professor Shen-lin Liu further puts forward that Pi-Xu (spleen deficient) and Yu-Du (stasis toxin) is the main pathogenesis of advanced GC. Based on the deficiency of spleen and vital qi, high viscosity of blood and formation of cancer plug are the important conditions for the recurrence and metastasis, while internal knot of blood stasis and toxin together with residual pathogenic factors are the key factors of GC. Therefore, to intervene in recurrence and metastasis, professor Liu proposes that inhibition of tumor thrombogenesis should be taken as the entry point of clinical TCM treatment and has developed Yiqi Huayu Jiedu decoction. In the prescription, Astragalus Root and Codonopsis Pilosula are used to strengthen Qi, Rhizoma Sparganii and Curcuma Zedoary are applied for dispersing blood stasis, which plays an important role in the prevention and treatment of postoperative recurrence and metastasis in GC.

Based on the above research foundation and progress, this prospective, multicenter, randomized controlled trial aims to evaluate the anti-recurrence and metastasis effect of Yiqi Huayu Jiedu decoction on postoperative GC. Moreover, we hope to form a treatment plan suitable for clinical promotion, and provide higher-level evidence-based medicine basis, thus laying a foundation for the development of guidelines for the treatment of GC with TCM.

## Author contributions

**Conceptualization:** Cun-En Wu.

**Formal analysis:** Wei-Wei Xue, Da-Wei Ding.

**Funding acquisition:** Peng Shu.

**Investigation:** Jin-Yong Zhou, Shen-Lin Liu, Rui-Ping Wang.

**Project administration:** Peng Shu.

**Supervision:** Shen-Lin Liu.

**Visualization:** Wei-Wei Xue, Yu-Wen Zhuang.

**Writing – original draft:** Wei-Wei Xue, Yu-Wen Zhuang.

**Writing – review & editing:** Cun-En Wu.
